# Circulating miRNAs Are Associated with Inflammation Biomarkers in Children with Overweight and Obesity: Results of the I.Family Study

**DOI:** 10.3390/genes13040632

**Published:** 2022-04-01

**Authors:** Fabio Lauria, Giuseppe Iacomino, Paola Russo, Antonella Venezia, Pasquale Marena, Wolfgang Ahrens, Stefaan De Henauw, Gabriele Eiben, Ronja Foraita, Antje Hebestreit, Yiannis Kourides, Dénes Molnár, Luis A. Moreno, Toomas Veidebaum, Alfonso Siani

**Affiliations:** 1 Institute of Food Sciences, National Research Council, 83100 Avellino, Italy; flauria@isa.cnr.it (F.L.); prusso@isa.cnr.it (P.R.); antovenezia@yahoo.it (A.V.); pask.mare@gmail.com (P.M.); asiani@isa.cnr.it (A.S.); 2 Leibniz Institute for Prevention Research and Epidemiology-BIPS, Achterstraße 30, 28359 Bremen, Germany; ahrens@leibniz-bips.de (W.A.); foraita@leibniz-bips.de (R.F.); hebestr@leibniz-bips.de (A.H.); 3 Department of Public Health and Primary Care, University Hospital 4K3 C. Heymanslaan, 10, 9000 Ghent, Belgium; stefaan.dehenauw@ugent.be; 4 Sahlgrenska Academy, University of Gothenburg, Medicinaregatan 3, 41390 Göteborg, Sweden; gabriele.eiben@medfak.gu.se; 5 Research and Education Institute of Child Health, 138 Limassol Ave, #205, Strovolos 2015, Cyprus; kourides@cytanet.com.cy; 6 Department of Pediatrics, Medical School, University of Pécs, H-7624 Pecs, Hungary; molnar.denes@pte.hu; 7 University of Zaragoza, Domingo Miral s/n, 50009 Zaragoza, Spain; lmoreno@unizar.es; 8 National Institute for Health Development, Hiiu 42, 11619 Tallinn, Estonia; toomas.veidebaum@tai.ee

**Keywords:** miRNAs, chronic low-grade inflammation, inflammation-associated biomarkers, overweight and obesity, children/adolescents, sex-related associations

## Abstract

Increasing data suggest that overnutrition-induced obesity may trigger an inflammatory process in adipose tissue and upturn in the innate immune system. Numerous players have been involved in governing the inflammatory response, including epigenetics. Among epigenetic players, miRNAs are emerging as crucial regulators of immune cell development, immune responses, autoimmunity, and inflammation. In this study, we aimed at identifying the involvement of candidate miRNAs in relation to inflammation-associated biomarkers in a subsample of European children with overweight and obesity participating in the I.Family study. The study sample included individuals with increased adiposity since this condition contributes to the early occurrence of chronic low-grade inflammation. We focused on the acute-phase reagent C-reactive protein (CRP) as the primary outcome and selected cytokines as plausible biomarkers of inflammation. We found that chronic low-grade CRP elevation shows a highly significant association with miR-26b-3p and hsa-miR-576-5p in boys. Furthermore, the association of CRP with hsa-miR-10b-5p and hsa-miR-31-5p is highly significant in girls. We also observed major sex-related associations of candidate miRNAs with selected cytokines. Except for IL-6, a significant association of hsa-miR-26b-3p and hsa-miR-576-5p with TNF-α, IL1-Ra, IL-8, and IL-15 levels was found exclusively in boys. The findings of this exploratory study suggest sex differences in the association of circulating miRNAs with inflammatory response biomarkers, and indicate a possible role of miRNAs among the candidate epigenetic mechanisms related to the process of low-grade inflammation in childhood obesity.

## 1. Introduction

During the past decade, obesity has become a global epidemic with substantial health, social, and economic implications. According to the World Health Organization, there are nearly 2 billion overweight adults worldwide and approximately 600 million of them are obese [1,2]. Numerous studies have recognized a strong association between an increased body mass index (BMI) and quality and expectancy of life given its interconnected effects with type 2 diabetes, hyperlipidemia, non-alcoholic fatty liver disease, hypertension, heart disease, stroke, arthritis, cancer, depression, asthma, psychological problems, and other non-communicable diseases [3,4,5].

Despite extensive investigation, most of the genetic variability in obesity remains unresolved and the influence of candidate genes in this context appears limited. Numerous studies have suggested that genes that predispose to obesity are not causal but act in conjunction with a variety of individual, environmental, and lifestyle factors, including obesogenic environments, low levels of education, sedentary habits, and reduced sleep hours, among others [6]. A highly energy-dense diet, as well as low physical activity levels, are considered driving factors [3]. Evidence also indicates that offspring born to obese mothers are prone to a higher BMI and fat accumulation, thus supporting the conceivable transgenerational risk contribution based on epigenetic mechanisms [7].

The basic tissue for energy storage in humans is the white adipose tissue. Aside from its storing function, this tissue is metabolically active. It releases hundreds of different factors, such as hormones including adiponectin and leptin, growth factors including Insulin-Like Growth Factor-1 (IGF-1) and Platelet-Derived Growth Factor (PDGF), and inflammatory mediators including Tumor Necrosis Factor-α (TNF-α), Interleukin-6 (IL-6), and Interleukin-8 (IL-8), all of which contribute to insulin sensitivity and dietary control. Growing data suggest that overnutrition-induced obesity triggers an inflammatory process in adipose tissue [8], with even moderate weight gain linked to inflammatory activation [9] and an upturn in the innate immune system [10]. Moreover, chemokines and their receptors also contribute to obesity development by activating the resident immune surveillance system [11]. This state is firmly interconnected to the local recruitment of macrophages and enhanced immune cell infiltration/proliferation/activation [12]. All these processes collectively point toward adipocyte hypertrophy and impaired adipogenesis [13]. The resulting persistent chronic low-grade inflammation represents a hallmark of obesity that leads to and perpetuates the state of metabolic alterations and insulin resistance in target organs, including the adipose tissue, liver, muscles, and vascular system [14]. Among the inflammatory biomarkers, the acute-phase reactant C-reactive protein (CRP) is considered the major factor associated with overweight and obesity in both adults and children [15,16].

In recent years, numerous biochemical players have been recognized as being involved in the inflammatory response, including microRNAs (miRNAs), small non-coding RNAs expressed in a wide variety of organs and cells and capable of potentially influencing almost all cellular functions. miRNAs are constituents of the epigenetic mechanisms that finely regulate the expression of messenger RNAs [17]. Until now, 2599 different miRNAs have been documented in humans (miRTarBase, release 8.0) [18]. Growing evidence also underlines their relevance as stable, non-invasive, and reliable biomarkers for a variety of pathophysiological conditions [19,20,21], including the body’s energy balance [22]. Many studies have established that altered miRNA profiles are interconnected with obesity [23] and other non-communicable diseases [24,25]. However, nowadays, epigenetics represents a critical but still poorly understood factor among the known molecular mechanisms related to the process of low-grade inflammation in obesity [26]. Among epigenetic players, miRNAs are progressively emerging as key regulators of immune cell development, immune responses, autoimmunity, and inflammation, capable of affecting both pro-and anti-inflammatory responses [26]. Indeed, by influencing definite signaling networks in the immune system, so-called immuno-miRs have been shown to impact both innate and adaptive immune responses in health and disease [27]. Of note, immuno-miRs may exert different functions in different cell types. Therefore, during inflammation, different cells undergo substantial transcriptional activation, thus presenting different sets of targets regulated by a given miRNA.

In this study, we aimed to identify the potential involvement of candidate miRNAs in relation to inflammation-associated biomarkers in a subsample of European children with overweight and obesity (OW/Ob) participating in the I.Family study (www.ifamilystudy.eu, last accessed on 21 February 2022) [28,29]. The primary outcome of this study was the association of miRNA expression with CRP. The association of miRNAs with the selected cytokines constituted a secondary outcome of the study. The study sample included individuals with OW/Ob since this condition is a known risk factor contributing to the early occurrence of chronic low-grade inflammation and interconnected metabolic changes.

## 2. Materials and Methods

### 2.1. Participants

The study was conducted on a subgroup of European children/adolescents with OW/Ob belonging to the I.Family study, an EC-funded project aiming at investigating determinants of food choice, lifestyle, and related health outcomes in children and adolescents of eight European countries (Belgium, Cyprus, Estonia, Germany, Hungary, Italy, Spain, and Sweden). A full description of the study designs, selection criteria, and participants’ characteristics, from which the subsample data are drawn, has been previously published [30]. A complete explanation of the I.Family study (registration number ISRCTN62310987) has been earlier reported [29]. The study was conducted according to the criteria set by the Declaration of Helsinki. Approval by the national ethics committees was obtained by each of the participating centers. Anthropometric characteristics, pubertal status, and dietary intake data were collected using standardized procedures; a full description of these methods has been previously published [31,32]. In each country, we first selected 20 children (n = 160) who retained overweight or obesity, i.e., who had a BMI z-score of more than + 1 at baseline and after 2 years at follow-up, respectively, and did not change more than ± 0.1 in BMI z-score per year (defined as overweight/obese) [33]. Out of 160 subjects, the current analysis was performed on a subsample of 79 overweight and obese children/adolescents (Belgium n = 7, Cyprus n = 6, Estonia n = 13, Germany n = 16, Hungary n = 8, Italy n = 12, Spain n = 5, and Sweden n = 12), with a complete dataset for the variables of interest, after the exclusion of 19 participants with hemolyzed samples.

### 2.2. Biochemical Analysis

The fasting venous blood draws were collected in BD Vacutainer^®^ blood tubes according to standardized operative procedures. A complete description of the sample collection and investigative procedures has been earlier published [34]. Clinical chemistry tests were determined as part of routine laboratory testing, in a central laboratory (University of Bremen, Centre for Biomolecular Interactions Bremen—CBIB). Serum samples stored at −80 °C were used to detect levels of CRP, Interleukin-1 Receptor Antagonist (IL1-Ra), IL-6, IL-8, Interleukin-15 (IL-15), and TNF-α using an electrochemiluminescent multiplex assay (using either single or MULTI-SPOT^®^ Assay Systems, Meso Scale Discovery).

### 2.3. miRNA Profiling

Taking advantage of the qPCR array technology, we previously reported that an altered circulating miRNA profile is associated with OW/Ob in children and adolescents [30]. In that study, we also identified four circulating miRNAs, hsa-miR-10b-5p (MIMAT0000254), hsa-miR-26b-3p (MIMAT0004500), hsa-miR-31-5p (MIMAT0000089), hsa-miR-576-5p (MIMAT0003241), potentially linked to increased CRP levels in subjects with OW/Ob (unpublished data). Of note, among the four miRNAs characterized, only hsa-miR-10b-5p was confirmed to be associated with OW/Ob. In the current investigation, we aimed to confirm the association of the candidate miRNAs with levels of CRP and the selected inflammatory biomarkers through validation by SYBR green-based real-time quantitative RT-PCR (RT-qPCR) in the new sample of children and adolescents with OW/Ob. Protocols for miRNA extraction and screening from plasma samples have been earlier published [30,32]. Individual plasma samples were first tested for hemoglobin levels and hemolyzed samples were excluded from the analysis [30]. Different assays were performed in triplicate employing the miScript Primer Assays according to the manufacturer’s instructions (Qiagen, Germany). miRNA relative levels were determined using the Cel-miR-39 spike-in as the endogenous normalizer [30]. Levels were calculated using the Data Assist v3.1 software package (Life Technologies, Thermo Fisher Scientific, Milan, Italy).

### 2.4. Statistical Analysis

Statistical analyses were achieved by using IBM SPSS Statistics software (v24.0. Armonk, NY, USA: IBM Corp.). Data collected were calculated as means and 95% confidence intervals (CIs). Associations of miRNA expression with CRP and the different interleukins were assessed using linear regression analyses, adjusting for covariates including country of residence, age, BMI *z*-score, pubertal status. Since potential sex disparities in CRP levels have been previously reported [35], we considered boys and girls separately in the statistical analysis. To control for the false discovery rate (FDR), the Benjamini–Hochberg (BH) method was adopted. The level of statistical significance was set at α < 0.05.

## 3. Results

### 3.1. Anthropometric Characteristics and Biochemical Markers of the Study Sample

The anthropometric and metabolic characteristics and levels of inflammatory markers of the 79 participants are reported in Table 1. There were no obvious differences regarding the anthropometric characteristics and tested biochemical markers between males and females.

### 3.2. RT-qPCR Validation in Individual Plasma Samples

After plasma extraction, the single candidate miRNAs were determined in individual assays by RT-qPCR. Differences in miRNA signatures with respect to anthropometric and biochemical variables were investigated. Associations of miRNA expression levels with CRP, the primary outcome of this study, and the selected interleukins were assessed using linear regression analysis stratified by sex. Results reported in Table 2 are adjusted for covariates including country of residence, age, BMI z-score, and pubertal status. CRP values show a significant association with miR-26b-3p and hsa-miR-576-5p exclusively in boys. Moreover, their associations with hsa-miR-10b-5p and hsa-miR-31-5p are highly significant only in girls. In Figure S1 is reported the distribution of selected miRNAs in relation to CRP levels in the girls’ and boys’ subgroups.

Table 3 reports the results of the associations of candidate miRNAs with the cytokines selected as additional inflammation biomarkers. A significant association of both hsa-miR10b-5p and hsa-miR-26b-3p with TNF-α, IL1-Ra, IL-8, and IL-15 levels was found exclusively in boys. No association of candidate miRNAs with IL-6 has been established. Moreover, none of the candidate miRNAs was associated with cytokine levels in girls.

## 4. Discussion

Inflammation is a physio-pathological process, commonly triggered by injuries and infections, and characterized by a complex flow of dynamically and finely regulated responses. The degree, the dynamics of pro-and anti-inflammatory networks, and the course of an inflammatory reaction may decisively impact the onset, progression, and development of health disorders. Inflammation and its supporting mechanisms are considered closely related to numerous diseases’ progression. Various studies have established that specific miRNAs participate in the development of innate and adaptive immunity, acting as crucial players in the fine-tuning of the inflammatory network. In this context, several miRNAs attenuate the response, while others, by depressing specific inhibitors, are capable of intensifying the immune reaction [36], with certain miRNAs essential for mounting the inflammatory response [37]. Notably, chronic inflammation is one of the main factors involved in the process of obesity progression [9,38], and several studies have reported that even limited weight gain is interconnected with the sustained inflammatory process [38].

Previously, we identified specific circulating miRNAs associated with childhood obesity in a subsample of the I.Family study [28,30]. Moreover, we also recognized in that study candidate miRNAs possibly related to CRP levels, among which only hsa-miR-10b-5p was associated with OW/Ob. In the present analysis, we report sex-related associations of these miRNAs with inflammatory biomarkers in children and adolescents with OW/Ob. Of note, the subjects enrolled for the current investigation belong to a different subgroup of the common I.Family population.

Given the different fat distribution and the influence of sex hormones, it is conceivable that the relationship between inflammatory markers and obesity may differ by sex. Accordingly, we stratified the study population by sex. Association analyses were corrected for confounding factors including the country of residence, age, BMI z-score, and pubertal status.

We found a clear indication pointing towards a cross-talk between candidate c-miRNAs and inflammatory biomarkers in the context of raised adiposity. Among the numerous inflammatory biomarkers, we focused on the acute-phase reactant CRP as the primary outcome of the study. CRP has been considered the strongest factor associated with OW/Ob in epidemiological studies [15]. Several reports consider CRP to be a consequence of an obesity condition rather than the cause [15]; conversely, increasing evidence establishes a causal role of CRP elevation in the onset and development of obesity by causing extensive tuning in the innate immune system and energy expenditure system [39].

We also found sex-related associations of the candidate miRNAs with the selected cytokines as plausible inflammation biomarkers. Except for IL-6, a significant association of both hsa-miR-26b-3p and hsa-miR-576-5p with TNF-α, IL1-Ra, IL-8, and IL-15 levels was found exclusively in boys. Moreover, no association of candidate miRNAs with cytokine levels was established in the girls’ subgroup.

Among the characterized miRNAs, miR-10b-5p is one of the first identified as abnormal in human cancer and, since its first description, it has been widely studied in this context. Recent papers also suggest that it participates in inflammation control and inflammation-associated diseases by regulating T cells [40,41]. Similarly, miR-26b has been described as a key regulator in carcinogenesis and cancer progression, acting as a tumor suppressor gene in several types of cancer. miR-26b has also been shown to play a role in inflammation as in cytokine secretion [42]. miR-26b also targets the inflammatory factor prostaglandin-endoperoxide synthase 2, which plays relevant roles in inflammatory diseases by inducing the production of prostaglandin E2 [43]. However, abnormal expression of miR-31-5p has been described in various cancers, where this miRNA plays a significant role in tumorigenesis, acting as either an oncogene or tumor suppressor, in a context-dependent manner, although the underlying mechanism remains unclear [44]. Moreover, miR-31 is involved in several inflammation-associated disorders. Interestingly, the molecular role of miR-31-5p activation in early inflammation has been recently defined [45]. Several studies have demonstrated that miR-576-5p acts as a tumor-promoting miRNA in several types of human tumors, highlighting its potential role as a predictor of cancer prognosis [46]. However, in silico studies have shown that miR-576-5p is involved in the regulation of inflammatory, growth, and proliferation signaling pathways.

Our apparently controversial results are in line with recent studies reporting sex influences on the severity and evolution of various inflammatory conditions [47,48]. Numerous studies have confirmed the role of sex hormones in the immune response and recent clinical data have shown significant sex differences in inflammatory markers also in prepubertal children, supporting a genetic contribution [47]. Sex differences occur in both innate and adaptive immune responses and are evolutionarily conserved across species [49]. Overall, there is accumulating evidence that sex is a critical variable that influences innate and adaptive immune responses, resulting in sex-specific outcomes, but the main molecular mechanisms remain elusive [48].

The findings of this exploratory study suggest major differences in the association of circulating miRNAs and inflammatory response biomarkers across sexes, pointing to a conceivable role of miRNAs among the candidate epigenetic mechanisms related to the low-grade inflammation process in childhood obesity, calling for more attention in this largely underexplored area. However, evidence concerning how these molecules may act remains questioned since the experimental design of our cross-sectional analysis, explorative in essence, cannot answer this question.

## 5. Conclusions

Many regulatory steps are relevant in the transformation and delivery of genetic information to cellular effectors. This network orchestration is finely regulated by both transcriptional and post-transcriptional mechanisms. Sex differences can provide substantial support in defining the course of these supervisory mechanisms [50]. The fascinating emergence of circulating miRNAs as stable and affordable molecules has opened up a promising opportunity for the identification of new crucial players in the onset and progression of inflammation in childhood obesity, as well as their potential application as non-invasive biomarkers. However, it is still uncertain whether the identified miRNAs are drivers of sex-related disparities in obesity-related inflammation or represent epiphenomena. Further molecular-oriented studies are needed to explore the functional relevance of the miRNA species identified.

## Figures and Tables

**Table 1:** Anthropometric and chemical characteristics of the study sample.

Ow/Ob	Boys (25/11)	Girls (31/12)
Age (years)	12.1 (11.5–12.8)	12.4 (11.9–12.9)
Puberty (% yes)	47.8	52.2
BMI *z*-score	1.8 (1.6–2.0)	1.7 (1.5–1.9)
CRP (mg/dL)	0.36 (0.08–0.63)	0.45 (0.14–0.77)
TNF-α (pg/mL)	4.3 (2.7–6.0)	3.9 (2.4–5.4)
IL-1Ra (pg/mL)	422.9 (339.7–506.2)	527.8 (398.0–657.6)
IL-6 (pg/mL)	1.2 (−0.1–2.5)	0.8 (0.5–1.0)
IL-8 (pg/mL)	38.5 (−23.1–100.0)	17.6 (1.7–33.6)
IL-15 (pg/mL)	2.6 (2.2–3.0)	3.0 (2.5–3.4)
hsa-miR-10b-5p	2.9 (2.1–3.6)	3.3 (2.7–3.9)
hsa-miR-26b-3p	2.4 (1.6–3.3)	2.7 (0.7–4.6)
hsa-miR-31-5p	1.1 (0.3–1.8)	1.5 (0.8–2.3)
hsa-miR-576-5p	7.3 (5.4–9.1)	6.4 (5.0–7.8)

Data are expressed as mean (CIs) or as frequency (%).

**Table 2:** Association of miRNA expression levels with CRP.

	Boys (36)	*q*-Value	Girls (43)	*q*-Value
hsa-miR-10b-5p	2.7 (1.7–3.7)	0.399	3.6 (3.0–4.2)	**0.008**
hsa-miR-26b-3p	3.2 (3.0–3.4)	**0.004**	2.1 (−0.9–5.0)	0.553
hsa-miR-31-5p	1.1 (−0.3–2.1)	0.914	1.3 (0.9–1.8)	**0.02**
hsa-miR-576-5p	8.3 (6.8–9.8)	**0.006**	6.8 (5.7–8.0)	0.187

Data are expressed as mean (CIs). Covariates: Country of residence, age, BMI z-score, pubertal status. *q*-values are BH-adjusted *p*-values. Values in bold indicate statistically significant results.

**Table 3:** Association of miRNA expression levels with selected cytokines (secondary outcome).

Cytokine	Sex	miR-10b-5p	*q*-Value	miR-26b-3p	*q*-Value	miR-31-5p	*q*-Value	miR-576-5p	*q*-Value
**TNF-α**	Boys	2.5 (2.0–3.0)	1.000	3.0 (2.6–3.4)	**0.006**	1.3 (0.3–2.2)	0.963	7.7 (6.4–9.0)	**0.005**
	Girls	3.2 (2.4–4.0)	0.635	2.4 (−0.4–5.2)	1.000	1.5 (1.0–2.1)	0.485	7.2 (6.1–8.2)	0.730
**IL1-Ra**	Boys	2.6 (2.1–3.1)	0.810	3.0 (2.6–3.4)	**0.005**	1.1 (−0.03–2.2)	0.393	7.9 (6.4–9.4)	**0.023**
	Girls	3.3 (2.5–4.1)	0.558	2.3 (−0.8–5.5)	1.000	1.6 (0.9–2.2)	1.000	6.6 (5.3–7.8)	0.735
**IL-6**	Boys	2.6 (2.0–3.2)	0.743	3.0 (2.1–4.0)	0.461	0.8 (−0.5–2.0)	0.963	7.1 (4.2–10.0)	0.932
	Girls	3.3 (2.5–4.1)	0.949	2.2 (−0.9–5.3)	0.732	1.6 (0.9–2.2)	0.692	6.8 (5.7–8.0)	0.625
**IL-8**	Boys	2.4 (1.8–3.0)	0.927	2.9 (2.5–3.3)	**0.005**	1.2 (0.2–2.2)	0.410	7.2 (5.6–8.9)	**0.021**
	Girls	3.3 (2.4–4.1)	0.649	2.5 (−0.5–5.4)	1.000	1.6 (1.1–2.1)	1.000	7.3 (6.2–8.4)	0.856
**IL-15**	Boys	2.6 (2.1–3.1)	0.588	2.9 (2.5–3.3)	**0.005**	1.2 (0.1–2.3)	0.890	7.5 (5.9–9.1)	**0.015**
	Girls	3.3 (2.6–4.0)	0.512	2.3 (0.9–2.3)	0.912	1.6 (0.9–2.2)	0.856	6.8 (5.7–8.0)	0.908

Data are expressed as mean (CIs). Covariates: Country of residence, age, BMI z-score, pubertal status. *q*-values are BH-adjusted *p*-values. Values in bold indicate statistically significant results.

## Data Availability

All data produced or analyzed during this study are included in this article.

## Supplementary Materials

The following supporting information can be downloaded at: https://www.mdpi.com/article/10.3390/genes13040632/s1, Figure S1. The different panels show the distribution of reported miRNAs in relation to CRP levels in girls and boys.
