# Supplementary material for: Circulating miRNAs Are Associated with Inflammation Biomarkers in Children with Overweight and Obesity: Results of the I.Family Study
Source: Genes (Basel). 2022 Apr 1;13(4):632. doi: 10.3390/genes13040632 (PMC9030192; doi:10.3390/genes13040632)
Supplement: Supplementary file 1 [file genes-13-00632-s001.zip › genes-1629745-supplementary.pptx]

## Slide 1
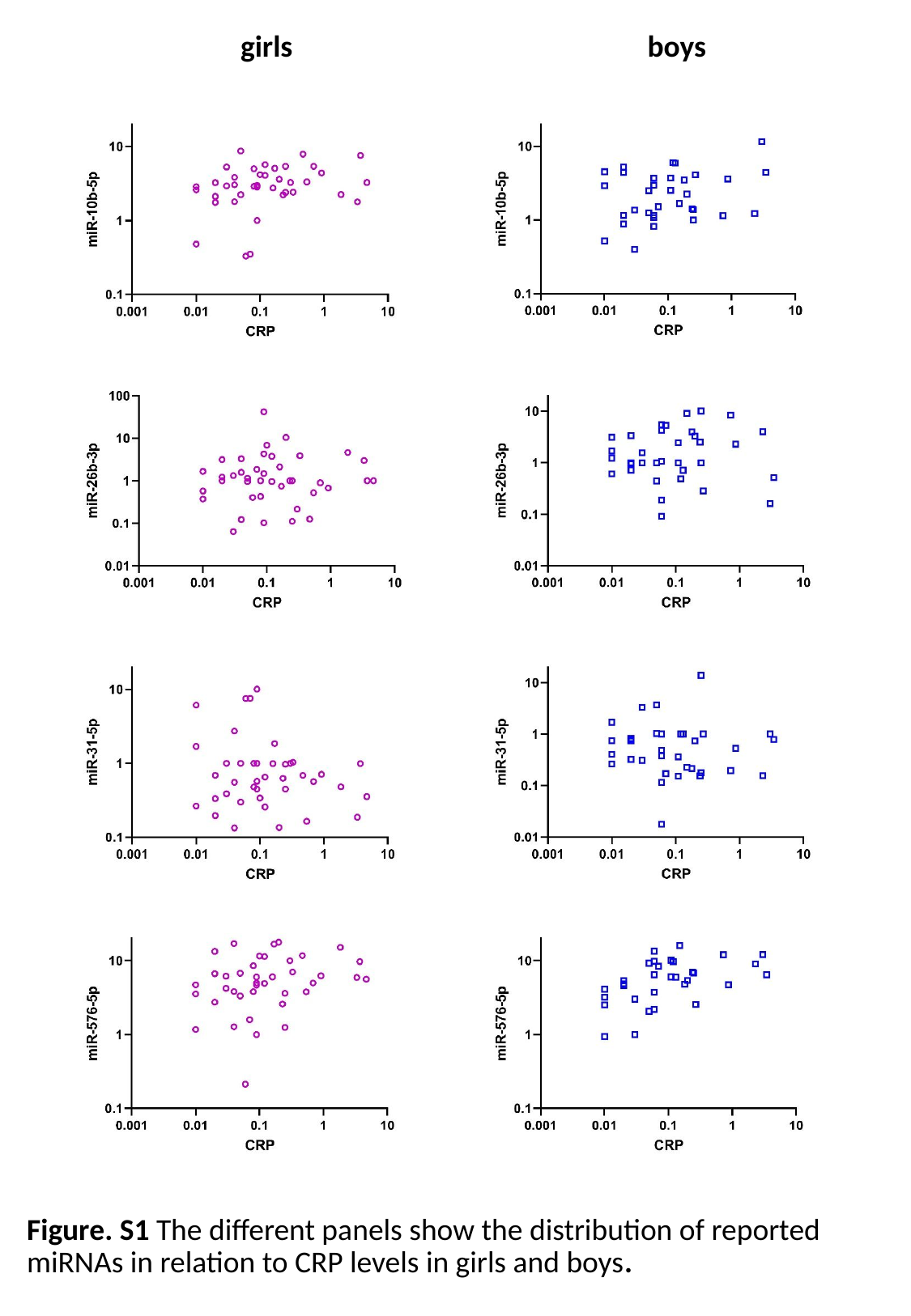

girls
boys
Figure. S1 The different panels show the distribution of reported miRNAs in relation to CRP levels in girls and boys.
